# Alzheimer-specific variants in the 3'UTR of Amyloid precursor protein affect microRNA function

**DOI:** 10.1186/1750-1326-6-70

**Published:** 2011-10-07

**Authors:** Charlotte Delay, Frédéric Calon, Paul Mathews, Sébastien S Hébert

**Affiliations:** 1 Centre de recherche du CHUQ (CHUL), Axe Neurosciences, Québec (Qc), Canada; 2 Université Laval, Faculté de médecine, Département de psychiatrie et de neurosciences, Québec (Qc), Canada; 3 Université Laval, Faculté de pharmacie, Québec (Qc), Canada; 4 NYU School of Medicine, Nathan Kline Institute, Orangeburg (NY), USA

**Keywords:** Amyloid precursor protein, microRNA, single nucleotide polymorphism, Alzheimer's disease, miR-147, miR-20a

## Abstract

**Background:**

APP expression misregulation can cause genetic Alzheimer's disease (AD). Recent evidences support the hypothesis that polymorphisms located in microRNA (miRNA) target sites could influence the risk of developing neurodegenerative disorders such as Parkinson's disease (PD) and frontotemporal dementia. Recently, a number of single nucleotide polymorphisms (SNPs) located in the 3'UTR of *APP *have been found in AD patients with family history of dementia. Because miRNAs have previously been implicated in APP expression regulation, we set out to determine whether these polymorphisms could affect miRNA function and therefore APP levels.

**Results:**

Bioinformatics analysis identified twelve putative miRNA bindings sites located in or near the *APP *3'UTR variants T117C, A454G and A833C. Among those candidates, seven miRNAs, including miR-20a, miR-17, miR-147, miR-655, miR-323-3p, miR-644, and miR-153 could regulate APP expression *in vitro *and under physiological conditions in cells. Using luciferase-based assays, we could show that the T117C variant inhibited miR-147 binding, whereas the A454G variant increased miR-20a binding, consequently having opposite effects on APP expression.

**Conclusions:**

Taken together, our results provide proof-of-principle that *APP *3'UTR polymorphisms could affect AD risk through modulation of APP expression regulation, and set the stage for further association studies in genetic and sporadic AD.

## Findings

AD is the most common form of dementia worldwide. Pathologically, the disease is defined by the intracellular accumulation of aggregated and hyperphosphorylated protein tau and the extracellular deposition of Aβ peptides, derived by proteolytic processing of APP. In genetic AD, mutations in the genes coding for APP, PSEN1 and PSEN2 lead to APP processing dysregulation resulting in Aβ over-production, accumulation and deposition, which ultimately leads to neuronal death [1]. Accumulating evidences also support the notion that increasing APP protein levels directly results in Aβ over-production [2], and that APP overexpression alone is sufficient to induce neurodegeneration an dementia [3-6].

miRNAs function as negative regulators of gene expression regulation, and play a critical role in neuronal function and survival [7]. These small (~21nt) non-coding RNAs interact with the 3'UTR of their target messenger RNA (mRNA) transcripts by partial sequence complementarity resulting in mRNA destabilization and/or translational inhibition [8,9]. This function is dependent on the miRNA seed region, comprising nucleotides 2-8 of the mature miRNA sequence. As changes in APP expression is intimately involved in AD development, several groups have now investigated the impact of miRNA modulation on APP expression. These studies identified a number of miRNAs capable of regulating APP expression *in vitro *and *in vivo*, including miR-20a, miR-17 (previously referred as miR-17-5p, http://www.mirbase.org), miR-106a, miR-106b, miR-101 and miR-16 [10-15]. Interestingly, miR-101, and miR-106b have been shown to be down-regulated in AD brain, therefore potentially contributing to increased APP expression and Aβ production [16,17].

Increasing evidence supports the hypothesis that genetic variants that either abolish existing miRNA binding sites or create illegitimate miRNA binding sites could contribute significantly to risk for neurodegenerative disorders. For instance, Wang *et al*. showed that a SNP located in the 3'UTR of the *fibroblast growth factor 20 (FGF20) *gene confers risk for developing PD, possibly by loss of miR-433 binding [18]. In addition, Rademakers *et al*. showed that increased binding of miR-659 to the 3'UTR of the *progranulin (GRN) *gene provides an important risk for TDP43-positive frontotemporal dementia [19]. More recently, Bettens *et al*. identified a number of AD-specific genetic mutations in the 3'UTRs of *APP *and *BACE1 *[20]. We extended these findings and established a detailed list of miRNAs with potential binding sites in or near polymorphisms located in the 3'UTR of human APP (hAPP) (Table 1). These include the *APP *variants T171C (unknown SNP ID), A454G (unknown SNP ID) and A833C (SNP ID rs3200120). These bioinformatics predictions were performed using logarithms available on-line, such as *Microcosm *[21], *TargetScan *[22] and *microRNA.org*[23]. In this study, we focused on polymorphisms predicted to abrogate (completely or partially) miRNA binding.

**Table 1:** Polymorphisms located in or near miRNA target sites located in the 3'UTR of *hAPP *

SNP ID	Position in 3'UTR	Polymorphism	Patient-specific	Predicted miRNA	Seed region
unknown	171	T/C	Y	hsa-miR-644	Y

				hsa-miR-147	N

				hsa-miR-323-3p	N

unknown	454	A/G	Y	hsa-miR-153	N

				hsa-miR-20a	N

				has-miR-17	N

				hsa-miR-106b	N

				hsa-miR-1245	Y

				hsa-miR-383	Y

rs3200120	833	A/C	unknown	hsa-miR-655	N

				hsa-miR-128	Y

				hsa-miR-199b-5p	Y

rs736479	914	G/A	Y	-	-

rs1059461	937	G/A	unknown	-	-

unknown	965	C/G	Y	-	-

rs45541739	967	G/A	unknown	-	-

The SNP ID, the nature of the polymorphism, as well as the patient specificity are indicated. The unknown SNP IDs can be found in Bettens *et al*. [20]. miRNAs which are predicted to interact with the 3'UTR of *hAPP *are listed, and it is noted when the SNP is located within the seed-region of the miRNA in question. Y = yes, N = no.

**Figure 1:**
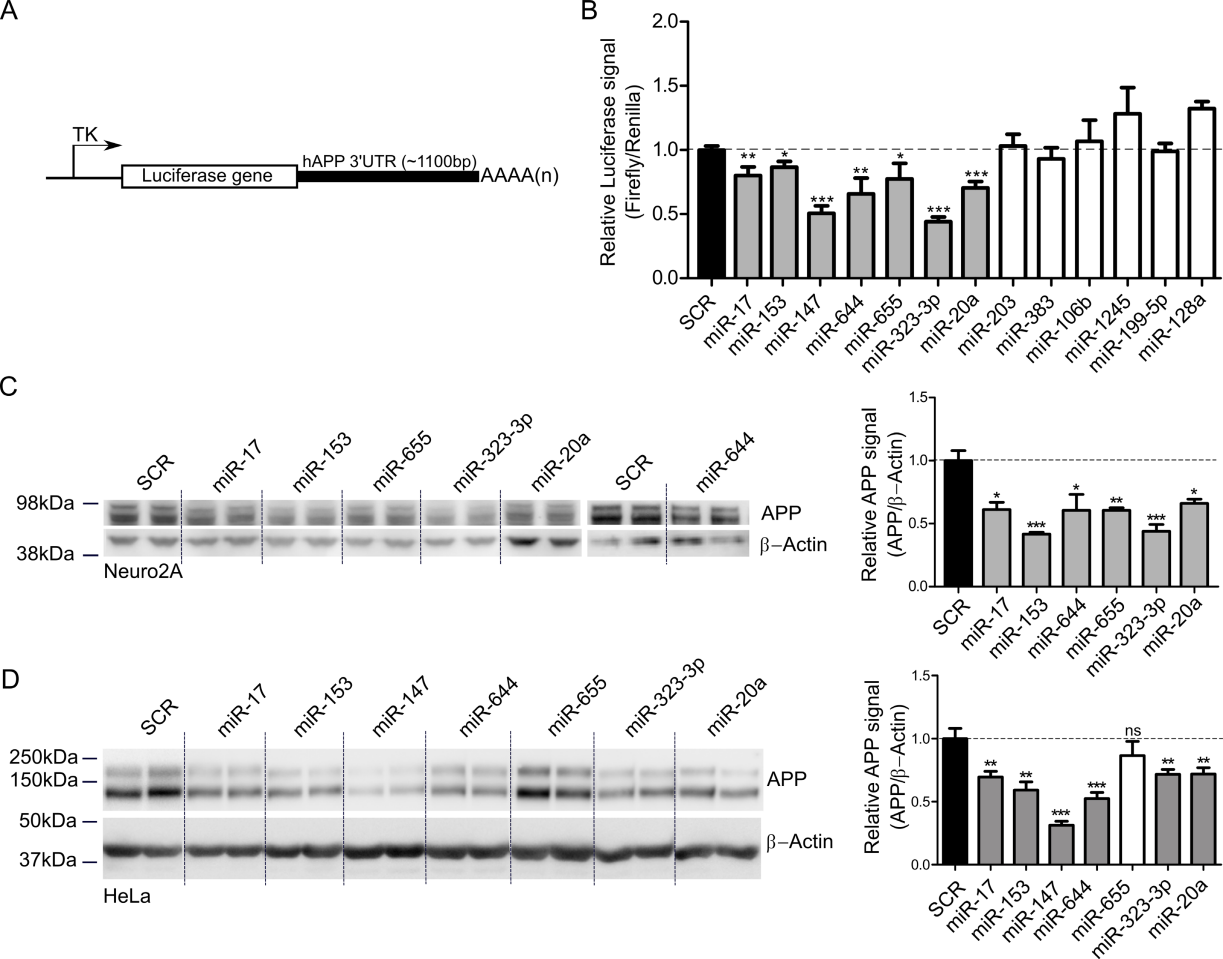
***In vitro *functional analysis of APP A**. Schematic representation (not to scale) of the luciferase reporter construct used in this study. TK; thymidine kinase promoter, AAAA(n); PolyA site **B**. HEK293T cells were transfected with 50nM pre-miRs (as indicated) as well as a reporter construct containing the 3'UTR of *hAPP*. The cells were lysed 24h post-transfection and luciferase signal was measured. Signals were normalization for transfection efficiency and graph represents the luciferase signals compared to the scrambled control (SCR). Statistical significance was assessed by Student paired t-test. (* = p < 0.05, ** = p < 0.01, *** = p < 0.001) **C. and D**. Neuro2A cells (C) and HeLa cells (D) were transfected with 50nM pre-miRNAs (as indicated). The cells were lysed 48h post-transfection and western blotting was performed. Representative (n = 3, in triplicate) western blots are shown. The ratios of the APP/β-Actin signals are presented. Measurements were normalized to the scrambled control (SCR). Statistical significance was assessed by Student's paired t-test. (* = p < 0.05, ** = p < 0.01, *** = p < 0.001).

We initially set out to determine whether candidate miRNAs could regulate APP expression. To this end, a luciferase construct harboring the *hAPP *full-length (~1100 bp) 3'UTR (Figure 1A) [10] was co-transfected with precursor miRNAs (pre-miRs) for miR-20a, miR-655, miR-147, miR-323-3p, miR-644, miR-203, miR-383, miR-106b, miR-153, miR-17, miR-128, miR-199b-5p, miR-1245 in HEK293 cells (Figure 1B). We used a scrambled miRNA sequence as negative control (SCR). As previously observed, miR-20a and miR-17 could significantly down-regulate luciferase (APP) reporter expression. We could not detect, however, a consistent effect of miR-106b on luciferase expression. Other miRNAs including miR-655, miR-147, miR-323-3p, miR-644 and miR-153 could negatively regulate luciferase expression, which is consistent with the bioinformatics predictions (Table 1). Taken together, these data suggest that miRNAs miR-20a, miR-17, miR-655, miR-147, miR-323-3p, miR-644 and miR-153 could be APP expression regulators. The candidate miRNAs that did not affect the luciferase signal, or increased its expression, were excluded in further analyses.

In order to validate these observations in more physiological conditions, we transfected our candidate pre-miRs into mouse neuroblastoma Neuro2A cells, a model previously used to study neuronal APP expression regulation by miRNAs [10]. Except for miR-147, each miRNA seed region was conserved between human and mouse *APP *3'UTRs [22]. For this reason, miR-147 was omitted from the mouse cell line experiments. In Neuro2A cells, all pre-miRs tested decreased endogenous APP expression when compared to the scrambled miRNA control (Figure 1C). We also transfected human HeLa cells with our candidate pre-miRs. All but one miRNA (miR-655) decreased endogenous APP expression when compared to the scrambled miRNA control (Figure 1D). Notably, miR-147 could efficiently down-regulate endogenous APP in these cells. Taken together, these data add to the growing list of miRNAs that could regulate endogenous APP expression in cultured cells, including neuronal-like cells. These include miR-147, miR-323-3p, miR-644 and miR-153, in addition to the previously identified miR-20a and miR-17.

**Figure 2:**
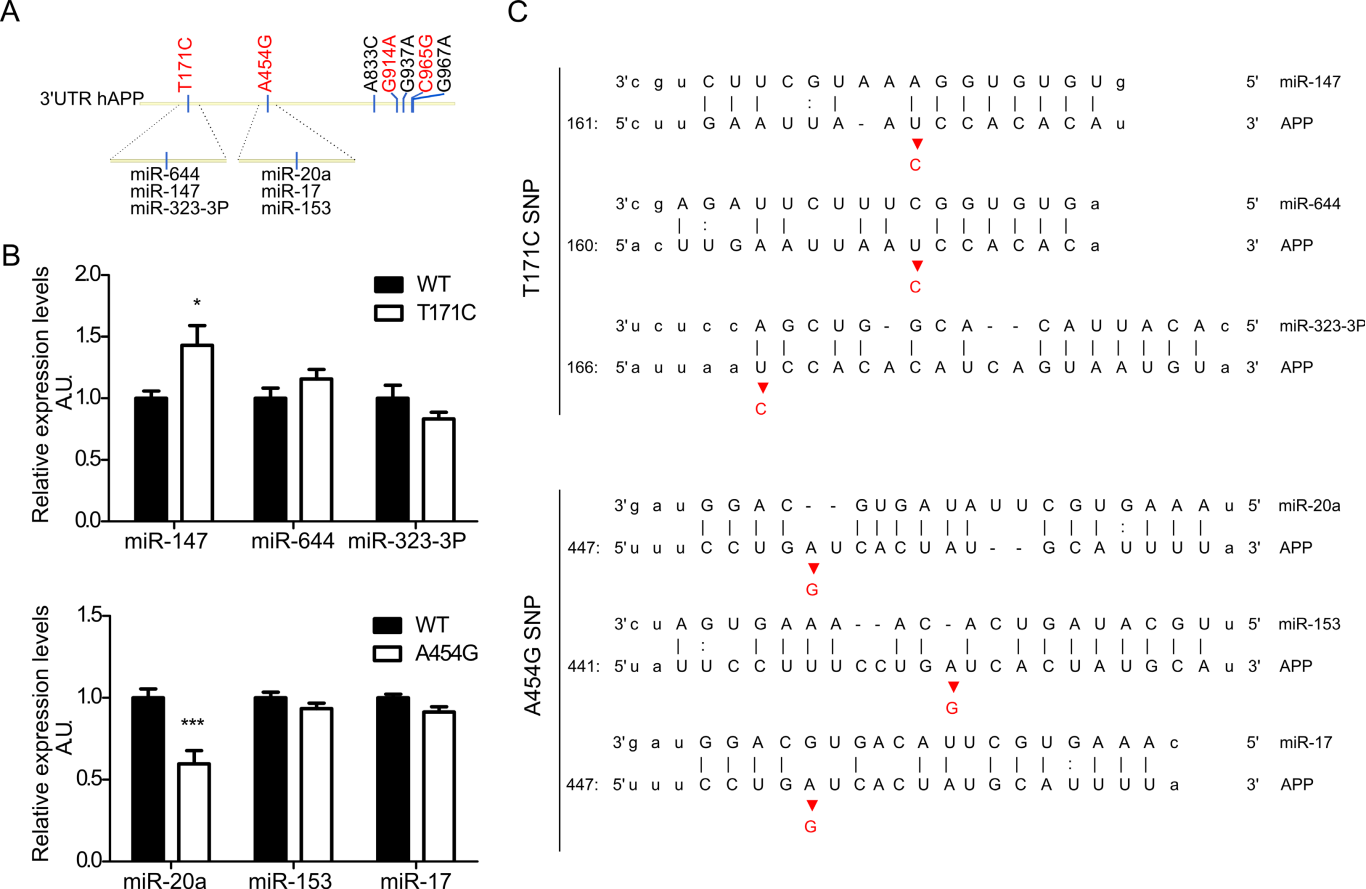
**SNPs are located near predicted miRNA binding sites on the *APP *3'UTR and affect the function of miR-147 and miR-20a A**. Schematic representation (not to scale) of SNP localization and predicted miRNA binding sites in the 3'UTR of *hAPP*. Red mutations represent the AD-specific SNPs, while the black mutations are not yet tested for their disease specificity. **B**. HEK293T cells were transfected with 5nM pre-miRs (as indicated) as well as a reporter construct containing the WT, T171C or A454G mutated 3'UTR of *hAPP*. The cells were lysed 24h post-transfection and luciferase signal was measured. After normalization for transfection efficiency, the signals were compared to the WT. Representative results (n = 3, performed in triplicate) are shown. Statistical significance was assessed by 2-way ANOVA (* = p < 0.05, ** = p < 0.01, *** = p < 0.001). SCR; scrambled, A.U.; Arbitrary Unit. **C**. Schematic representation of base pair matching between miRNAs and the 3'UTR of *hAPP*. The seed region of the miRNAs is indicated. The red bases represents the SNPs T171C (upper panel) or A454G (lower panel).

We next tested whether the "APP-positive" miRNAs were affected by the *APP *3'UTR polymorphisms (Table 1). For these experiments, we focused on AD-specific SNPs, and generated *hAPP *3'UTR luciferase constructs with T171C or A454G mutations. Our screens indicated that miR-147, but not miR-644 or miR-323-3p, was significantly affected by the T171C mutation when compared to the wild-type (WT) construct (Figure 2B, upper panel). In a similar set of experiments, we could show that miR-20a, but not miR-153 or miR-17, was affected by the A454G mutation (Figure 2B, lower panel). As expected, miR-147 is a less potent inhibitor of APP expression in the presence of T171C. On the other hand, and surprisingly, miR-20a further decreased the expression of APP in the presence of A454G. For miR-147, the effect may be explained by the fact that T171C is located immediately adjacent to the miR-147 seed region, therefore directly inhibiting miRNA binding (Figure 2C, upper panel). This effect can also be explained as the mutation increased the binding energy (ΔG = -18.9 kCal/mol *vs*. -16.8 kCal/mol) between miR-147 and the 3'UTR of *hAPP*, which makes the binding less favorable. Although we observe a net gain of function of miR-20a towards the SNP A454G form compared to the WT 3'UTR, A454G is not predicted to change the binding energy (ΔG = -22.8 kCal/mol) between miR-20a and the 3'UTR of *hAPP*, and the SNP is also not located within the seed region (Figure 2C, lower panel). Taken together, we identified two miRNAs, that is, miR-147 and miR-20a, affected by AD-specific 3'UTR SNPs.

In conclusion, we provide evidence for the first time that polymorphisms located in the 3'UTR of *hAPP *may affect its expression, at least in the experimental conditions tested here. Indeed, we show that two AD-specific 3'UTR variants previously identified by Bettens and colleagues [20] affect the modulating activity of miR-147 and miR-20a on the expression of APP. SNP T171C decreases the ability of miR-147 to down-regulate APP, theoretically leading to increased APP and Aβ production. On the other hand, SNP A454G increases the effect of miR-20a, suggesting that APP expression is reduced in these patients. Although these data seem to contradict with the main hypothesis that increased APP levels lead to AD, some reports indicate that decreasing the APP levels might have deleterious consequences in the brain [24,25]. Another possibility is that miR-20a levels (or function) vary depending on brain region or disease state, therefore only locally affecting APP. In line with this hypothesis, our preliminary data suggest that certain "APP-positive" miRNAs are differently expressed between human regions (Delay *et al*., not shown). Finally, we cannot exclude at this stage of investigation that the second, less functional miR-20a binding site located at position 709-715 in the *hAPP *3'UTR [10], could become more prone to miRNA regulation in the presence of SNP A454G. While follow-up studies are required to evaluate the incidence of these variants in other populations, our results suggest that 3'UTR mutations may contribute to risk for AD development. These studies also set the stage for validation studies regarding APP expression regulation by specific miRNAs *in vivo *in the brain, and further evaluation of 3'UTR variants in AD-related genes in general.

## Methods

### Cell culture

Human HEK293 and HeLa cells, as well as mouse Neuro2A cells, were cultured in DMEM medium (Invitrogen, Carlsbad, CA, USA) supplemented with 10% heat-inactivated fetal bovine serum. One day before transfection, HEK293 cells were plated at 100,000 cells per well in 24-well plates, Neuro2A cells at 192,000 cells per well in 6-well plates, while HeLa cells were plated at a 20% confluence in 6-well plates. Transfection was performed using Lipofectamine 2000 (Invitrogen, Carlsbad, CA, USA) according to the manufacturers instructions.

### cDNA constructs

The full-length *hAPP *3'UTR luciferase construct was described previously [10]. Mutagenesis was performed by TOPgene technologies (Montreal, Quebec, Canada) and validated by sequencing.

### Luciferase assay and protein analysis

Cells were transfected with 5 or 50 nM (see text) pre-miRs (Applied Biosystems, USA), 2.5 ng/cm^2 ^pRL control vector, and 50ng/cm^2 ^pGL3_HSV TK_3'UTR *hAPP *WT or T171C or A454G plasmids. Twenty-four hours post-transfection, cells were lysed, and luciferase activity was measured according to the manufacturer's instructions (Promega, USA). For western blots, cells were lysed in RIPA buffer [50mM Tris Hcl, 1% NP40, 0.9% NaCl, 0.25% Na-deoxycholate, 1mM EDTA, 1x proteinase inhibitors (Roche, Basel, Switzerland), 1mM PMSF, 1mM Na3VO4 and 1mM NaF], mixed with LDS sample buffer (Invitrogen, Carlsbad, CA, USA) containing 5% beta-mercapto-ethanol and boiled at 95°C for 8 min. Crude protein lysates (10 μg) were immunoblotted with the APP C1.61 (for human APP), the APP C-ter (Sigma Aldrich, St-Louis, MO, USA) (for mouse APP) or β-Actin (Sigma Aldrich, St-Louis, MO, USA) antibodies, and detected using the ECL detection kit (Millipore, Billerica, MA, USA). Quantifications were performed using the Multi Gauge software (FUJIFILM, Minato-ku, Tokyo, Japan).

### Statistics

Statistical significance of western blots and luminescence quantifications were determined using 1-way ANOVA, 2-way ANOVA or Student's paired t-test as indicated in the text. Calculations were made using the GraphPad Prism 5 software.

## List of Abbreviations

Amyloid precursor protein, APP; single nucleotide polymorphism, SNP; 3'UTR; 3'untranslated region, nt; nucleotide;

## Competing interests

The authors declare that they have no competing interests.

## Authors' contributions

CD and SH participated in the design of the study. CD performed the experiments and statistical analysis. PM and FC provided material. CD and SH wrote the manuscript. All authors read and approved the final manuscript.
